# Quantifying the effects of plant density on soybean lodging resistance and growth dynamics in maize-soybean strip intercropping

**DOI:** 10.3389/fpls.2023.1264378

**Published:** 2023-11-23

**Authors:** Li Wang, Bin Cheng, Tao Zhou, Shuzhong Jing, Ranjin Liu, Yang Gao, Chaoyang Deng, Wenwei Ye, Zhigang Luo, Ali Raza, Mei Xu, Wenyan Wang, Weiguo Liu, Wenyu Yang

**Affiliations:** ^1^ Key Laboratory of Crop Ecophysiology and Farming System in Southwest China, Ministry of Agriculture, Sichuan Agricultural University, Chengdu, China; ^2^ Sichuan Engineering Research Center for Crop Strip Intercropping System, Sichuan Agricultural University, Chengdu, China; ^3^ Chengdu Damei Seed Industry Co., Ltd., Chengdu, China; ^4^ State Key Laboratory of Southwestern Chinese Medicine Resources, Chengdu University of Traditional Chinese Medicine, Chengdu, China; ^5^ Sichuan High-end Talent Center, Chengdu, China; ^6^ CAS Key Laboratory of Mountain Ecological Restoration and Bioresource Utilization & Ecological Restoration and Biodiversity Conservation Key Laboratory of Sichuan Province, Chengdu Institute of Biology, Chinese Academy of Sciences, Chengdu, China

**Keywords:** soybean, intercropping, planting density, growth function, lodging resistance index

## Abstract

Shading-induced soybean stem lodging is a prevalent concern in the maize (*Zea mays L*.)-soybean (*Glycine max L. Merr*.) strip intercropping system, leading to a substantial decline in yield. Nevertheless, the associations between soybean growth, stem lodging, and yield formation in this scenario remain unclear. To investigate this, the logistic and beta growth models were utilized to analyze the growth process of soybean organs (stems, leaves, branches, and pods) and the accumulation of carbohydrates (lignin, cellulose, and sucrose) at three planting densities (8.5, 10, and 12.5 plants m^−2^) in both strip intercropping and skip strip monoculture systems. The results indicate that shading stress caused by maize in the intercropping system reduced lignin and cellulose accumulation in soybean stems, thus decelerating soybean organ growth compared to monoculture. Furthermore, intercropped soybean at higher planting density (PD3) exhibited a 28% reduction in the maximum dry matter growth rate (*c_m_*) and a 11% decrease in the time taken to reach the maximum dry matter growth rate (*t_e_*) compared to the lower planting density (PD1). Additionally, a 29% decrease in the maximum accumulation rate (*c_max_*) of sucrose, lignin, and cellulose was observed, along with a 13% decrease in the continuous accumulation time (*t_c_*) of these carbohydrates in intercropped soybean at PD3. Interspecific and intraspecific shading stress led to a preferential allocation of assimilates into soybean stems, enhancing plant height during the initial stage, while at later stages, a greater proportion of sucrose was allocated to leaves. Consequently, this hindered the conversion of sucrose into lignin and cellulose within the stems, ultimately resulting in a reduction in the lodging resistance index (*LRI*). Overall, this study provides valuable insights into the effects of shading stress on soybean growth and yield. It also emphasizes how optimizing planting density in intercropping systems can effectively alleviate shading stress and enhance crop productivity.

## Introduction

Sustaining the rapidly expanding global population with limited land resources presents a major challenge for humanity (Digrado et al., 2020; Chapman et al., 2022). Intercropping is a promising strategy to address the challenge of feeding a growing population with limited resources by improving land use and light energy efficiency (Martin-Guay et al., 2018; Raza et al., 2019b; Zeng et al., 2019; Feng et al., 2020; Kang et al., 2020). Maize (*Zea mays L*.)-soybean (*Glycine max L. Merr*.) strip intercropping, being one of the most popular intercropping systems, allows for the preservation of maize yield while also obtaining an additional soybean harvest (Liu et al., 2018b; Feng et al., 2020; Raza et al., 2021). However, the maize soybean strip intercropping system often exhibits a considerable reduction in soybean yield compared to monoculture soybean. Numerous studies attribute this outcome primarily to the shading effect of maize on soybean (Liu et al., 2016; Raza et al., 2020). Moreover, the shading effect of maize on soybean diminishes photosynthate synthesis and disturbs assimilate allocation, consequently resulting in soybean stem lodging and subsequent yield decline (Yang et al., 2014; Hussain et al., 2020a; Yang et al., 2020). Studies indicate that the lodging resistance of lower crops in intercropping systems depends on organ development, coordination, and the accumulation of lignin and cellulose in stems (Mao et al., 2018; Hussain et al., 2020a; Zhang et al., 2020). Plant morphogenesis is significantly influenced by assimilate transport and partitioning, which, in turn, are affected by planting patterns, including planting densities and arrangements (Patrick et al., 2001).

Intercropping and high-density planting are widely adopted practices worldwide to enhance crop production. Nevertheless, when sunlight is obstructed by tall vegetation, a substantial amount of red and blue light is absorbed by the higher plant canopy for photosynthesis. Consequently, the reduction in red and blue light due to shading is more pronounced compared to light of other wavelengths. The yield potential of these planting patterns is always limited by the unfavorable shade avoidance syndrome (SAS), which is caused by the decreased red and blue light (Libenson et al., 2002; Wang et al., 2016; Liu et al., 2018b). Plants perceive shading through photoreceptors, including phytochromes (PHYs) and cryptochromes (CRYs) (Fraser et al., 2016; Liu et al., 2021). PhyB in *Arabidopsis* primarily mediates the shade avoidance response induced by a low red to far-red light ratio (Ballaré, 1999). The blue light receptors, CRYs, are responsible for sensing low blue light (LBL) shade signals. The loss-of-function CRYs or deprivation of blue light induces a SAS that shares many similarities with plant growth under low R:FR conditions (Keller et al., 2011). However, *phyB* and *cry* mutants retain their responses to LBL and low R:FR, and LBL can enhance low R:FR-induced SAS, indicating that PHY and CRY mediate SAS through distinct pathways (Keller et al., 2011; de Wit et al., 2016). Recent research has found that LBL, rather than low R:FR treatment, causes excessive elongation of soybean stems, indicating that *GmCRYs* play a crucial role in mediating LBL-induced SAS (Lyu et al., 2021). Moreover, it has been demonstrated that the involvement of gibberellin (GA) homeostasis in LBL-induced stem elongation in soybean (Lyu et al., 2021). Other plant hormones such as auxin are also involved in the regulation of shade avoidance in soybean (Jiang et al., 2020). The application of auxin transport inhibitors can completely block the elongation of the hypocotyl in *Arabidopsis* induced by low R:FR conditions but does not fully suppress hypocotyl elongation in *Arabidopsis* under low blue light (LBL) conditions. Therefore, it suggests that low R:FR and LBL-induced shade avoidance responses in plants may be mediated through different mechanisms (Pedmale et al., 2016). Moreover, the elongation of the hypocotyl in *Arabidopsis* under shaded conditions requires the involvement of brassinosteroids. The hypocotyl of the brassinosteroids synthase mutant *diminuto/dwarf1* cannot exhibit typical shade avoidance response characteristics when plants are under shade environment (Luccioni et al., 2002). Overall, the shade avoidance response of the hypocotyl requires the collective involvement of auxin, gibberellins, and brassinosteroids. Light signals regulate the levels of hormones and signal transduction within cells to control plant stem elongation (Wang et al., 2013).

The shade avoidance response is an adaptive growth mechanism in plants in response to the light environment, but some of these trait changes can be disadvantageous for agricultural production. As plants grow, alterations in the light environment trigger a range of characteristic shade avoidance responses in soybean, including stem and petiole elongation, reduced stem diameter and branching (Nacer et al., 2012; Yang et al., 2014; Wu et al., 2017b). Chlorophyll fluorescence, as a sensitive probe in photosynthesis research, can effectively reflect the impact of environmental stressors on photosynthesis (Huang et al., 2011). Research indicates that shading leads to a reduction in the rate of photosynthetic electron transfer in the photosystem II (PSII) reaction centers of soybean leaves, resulting in an accumulation of excess light energy in the PSII reaction centers (Fan et al., 2017). Shading resulted in a decrease in the light saturation point (LSP), the light compensation point (LCP), and the maximum photosynthetic rate (*Pnmax*), while increasing the apparent quantum yield (AQ) (Hussain et al., 2020b). To maintain a higher level of energy metabolism, soybean leaves enhance photochemical reaction efficiency by reducing heat dissipation. In order to maximize light capture in shaded environments, carbon allocation is shifted toward stem elongation, resulting in compromised leaf and root development and subsequently impacting photosynthesis and nutrient uptake (Gommers et al., 2013; Wu et al., 2017b). The SAS in soybean diverts resources from agronomically significant tissues to facilitate stem elongation, thereby causing lodging and potentially resulting in a reduction of soybean yield by up to 22% (Noor and Caviness, 1980). The primary limitation to high-density planting and intercropping arises from the shade-induced imbalance in dry matter allocation between stems and other tissues, consequently affecting soybean yield. Therefore, understanding the assimilation strategy and allocation patterns in various planting densities and patterns can be an effective approach to enhance the allocation of resources to stems and pods, thereby improving lodging resistance and reducing yield losses.

Cellulose and lignin impart structural integrity to the plant cell wall, enabling it to withstand external pressure and maintain plant morphology (Somerville, 2006; Yeats et al., 2016). Accumulation of lignin and cellulose strengthens the mechanical properties of stems, thereby enhancing lodging resistance in plants (Wu et al., 2017a; Zheng et al., 2017). In intercropping systems, elevated cellulose and lignin concentrations in the cell wall confer improved stem mechanical strength and reduced lodging rates (Liu et al., 2016; Hussain et al., 2020a). Studies have demonstrated that soybean varieties with elevated concentrations of cellulose, lignin, and associated enzyme activities in the stem display enhanced lodging resistance in intercropping systems compared to shade-susceptible soybean varieties (Liu et al., 2016; Liu et al., 2018a). Sucrose is not only one of the main photosynthates, but a form of photosynthate transported from leaves to other organs (Pego et al., 2000; Zhu et al., 2019). The activities of sucrose phosphate synthase and sucrose synthetase in the stem are associated with shade tolerance and lodging resistance of soybean in the intercropping systems (Liu et al., 2016). Research has shown that sucrose synthase catalyzes the reversible conversion of uridine diphosphate (UDP) and sucrose into uridine diphosphate glucose (UDP-Glc) and fructose, with sucrose metabolism being the primary source of UDP-Glc for cellulose synthesis (Ruan, 2014). Additionally, sucrose metabolism plays a critical role in lignin synthesis through various metabolic pathways, including the shikimic acid pathway, phenyl propionic acid metabolic pathway, and specific lignin synthesis pathway (Boerjan et al., 2001). Furthermore, a previous study observed that intercropped soybean at the seedling stage exhibited elevated sucrose concentration in stems but lower cellulose levels compared to monoculture soybean, primarily attributed to shading-induced inhibition of sucrose conversion into cellulose (Liu et al., 2016). Therefore, it is crucial to understand how planting density, particularly shading from maize in intercropping, influences the accumulation of lignin and cellulose in soybean stems and the subsequent impact of these changes on lodging.

Unlike maize-soybean relay strip intercropping system, crops in the strip intercropping system are sown and harvested at the same time (Liu et al., 2017; Liu et al., 2018b). Soybean in the above-mentioned two planting patterns experience two different light environments. In the strip intercropping system, soybean experiences shading by maize during the middle and later stages of growth, whereas in the relay strip intercropping system, soybean encounters shading during the seedling stage (Yang et al., 2014; Liu et al., 2017; Cheng et al., 2020). Soybean in both of these planting patterns experiences yield loss due to lodging (Cheng et al., 2020; Hussain et al., 2020a). The majority of studies investigating stem lodging resistance have primarily concentrated on soybean in the relay strip intercropping system, leaving limited understanding of soybean in the strip intercropping system (Deng et al., 2016; Liu et al., 2016; Raza et al., 2019a; Hussain et al., 2020a). In fact, achieving a balance between resource allocation for stem mechanical support, leaf photosynthetic production, and pod yield formation becomes increasingly complex for soybeans in the strip intercropping system during the later stages of growth (under shading conditions).

The daily expansion of an organ occurs through the distribution of growth substrates, influenced by various environmental and physiological factors (Yin et al., 2003). However, organ dry matter measurements are often restricted to specific time points during the growth period, usually corresponding to distinct phenological stages. Obtaining direct measurements of the organ biomass partitioning index, which represents the proportion of daily organ growth rate to the daily plant growth rate, is not feasible solely through field experiments. Growth functions, including the beta and logistic growth functions and their derivatives, have been widely used to describe the daily accumulation of dry matter in diverse systems, and these functions can also be utilized to derive allocation functions for assimilates (Yin et al., 2003; Bai et al., 2016; Mao et al., 2018; Zhang et al., 2020). The logistic growth function is suitable for scenarios where material continues to increase, whereas the beta growth function explicitly allows for a decline in biomass beyond *W_max_*. The growth function’s three parameters possess distinct biological interpretations: the maximum dry matter (*W_max_*), the time when *W_max_* is reached (*t_e_*), and the time at which maximum growth rate is reached (*t_m_*). The growth rate can be obtained by fitting the function to periodically collected biomass data and calculating its first derivative. This approach enables the determination of the partitioning index by comparing the growth rates of different organs under varying environmental conditions and agricultural practices (Dong et al., 2017; Mao et al., 2018). Hence, the study utilized the beta and logistic growth functions to simulate the dynamics of sucrose, lignin, cellulose, and dry matter accumulation in soybean with varying degrees of lodging by implementing different densities.

In the maize soybean strip intercropping systems, soybean frequently undergoes lodging and subsequent yield losses caused by shading from maize. In this system, both crops are simultaneously planted, and soybeans are subjected to increasing shading from maize, creating a dynamic light environment. However, our comprehension of the physiological processes related to material production, transport, and allocation in soybeans under such conditions is limited. The aim of this study was to quantify the assimilate allocation of soybean in the maize-soybean strip intercropping system in response to plant density. This was achieved by analyzing biomass data, fitting growth functions, and deriving partitioning functions based on the fitted models. Additionally, we conducted a comparative analysis of the impacts of planting densities on the accumulation dynamics of cellulose and lignin in stems and the allocation strategies of dry matter in different organs. This analysis utilized the logistic and beta growth functions, along with their derivative partitioning functions. Consequently, we elucidated the growth, stem lodging resistance, and yield of soybean in the strip intercropping system in relation to planting density.

## Materials and methods

### Experimental site

Field experiments were conducted from 2019 to 2020, at the Chongzhou Experimental Farm of Sichuan Agricultural University, Sichuan Province, China. The climate of the experimental area is humid and subtropical. The annual mean air temperature is 15.9 °C, of which the mean air temperature of the hottest month (July) is 25 °C, and the coldest month (January) is 5.4 °C (**Supplementary Figure 1**). The annual mean sunshine hours and the annual mean rainfall are 1161.5 h and 1012.4 mm, respectively. The soil at the experimental site is classified as clayey soil, with total nitrogen 1.6 g kg^-1^, total phosphorus 1.3 g kg^-1^, total potassium 15.2 g kg^-1^, available nitrogen 299.5 mg kg^−1^, available phosphorus 1.3 g kg^-1^, available potassium 169.4 mg kg^−1^, organic matter content of 24.3 g kg^−1^ and the pH of the top 0-20 cm soil layer is 7.1.

### Experimental material and design

In this experiment, Zhenghong-505 (semi-compact), a maize cultivar and Chuandou-16 (shade-sensitive), a soybean cultivar, which are mainly planted in southwest China, were used as materials. The experiment was a two-factor randomized block design with three replications. The planting patterns (e.g. maize-soybean strip intercropping system and skip strip monocropping system) were as the main factor, and three soybean planting densities (e.g. PD1 = 8.5 plants m^-2^, PD2 = 10 plants m^-2^ and PD3 = 12.5 plants m^-2^, thereafter abbreviated as PD1, PD2 and PD3 respectively) were as the secondary factor. The classic wide and narrow rows planting with 2 m of each strip width and 6 m of each strip length was adopted by the maize-soybean strip intercropping system. The area of each individual plot was 36 m^2^ (6.0 m×6.0 m) for both strip intercropping and skip strip monocropping. Each individual intercropping plot consisted of 3 maize-soybean strips, and skip strip monocropping plot included three maize or soybean strips. As shown in **Supplementary Figure 2**, in the maize-soybean strip intercropping system, two rows of maize were planted in narrow row spacing of 40 cm, with planting space of 20 cm (i.e. 5 plants m^-2^), and two rows of soybean were planted in wide row spacing of 160 cm, with planting space of 8 cm, 10 cm and 12 cm (i.e. PD3, PD2 and PD1 respectively) (**Supplementary Figures 2A, C**). In the sole soybean system with skip strips, the planting method was the same as that of intercropped soybean without maize strip, which served as the control (**Supplementary Figures 2B, D**). Both maize and soybean were sown on 2 April in 2019 and 2020, and harvested on 26 July and 2 August in 2019 and 2020, respectively.

All maize strips were treated with compound fertilizer (N: P: K = 15: 15: 15) at 80 g m^-2^ as the base fertilizer before sowing. The urea (N ≥ 46%) was applied as fertilizer with 7.8 g m^-2^ at the jointing of maize and the second dose of urea (N ≥ 46%) was applied as fertilizer with 13.2 g m^-2^ at heading stages of maize (Cheng et al., 2020). Whereas there was no fertilizer application in all soybean strips due to the fertile soil in the experimental area. In addition, weeding, pest control and irrigation are used in the daily management of maize and soybean.

### Data collections

The lodging resistance index (*LRI*), dry matter growth of soybean (i.e. stems, leaves, branches and pods) and accumulation of sucrose, cellulose and lignin were measured 5 times every 14 days from 35 to 91 days after sowing in 2019 and 2020, respectively. At the mature stage of soybean and maize, soybean seeds and maize cobs were collected respectively for grain yield measurement in 2019 and 2020.

### Measurements

#### Lodging percentage and lodging resistance index

The lodging percentage (*LP*, Eqn. 1) was determined by randomly inspecting each soybean strip that was not sampled under different treatments (Xiang et al., 2016).


(1) $$LP\left(\%\right)=\frac{TNLP}{TNP}*100$$

Where *LP* (%) is the lodging percentage, *TNLP* is the total number of plants lodged in a plot, *TNP* is the total number of plants in a plot.

Lodging resistance index (*LRI*, Eqn. 2), as an indicator of soybean stem strength, was calculated by the previous method (Cheng et al., 2020; Hussain et al., 2020a).


(2) $$LRI=\frac{SBF}{MSL*AGW}$$

Where *LRI* represents the lodging resistance index, *SBF* represents the stem bending force, *MSL* represents the main stem length, *AGW* represents the above ground biomass fresh weight.

### Measurements of maize and soybean plant height and maize-crown breadth

To determine the effect of the light environment of soybean in the maize soybean strip intercropping system, we measured the plant height of soybean and maize, as well as the breadth of maize crown, using a ruler with a measuring range of 0 to 3.0 m and an accuracy of 0.1 cm during the period from 35 to 91 days after sowing. These measurements were taken every 14 days, for a total of 5 times.

### Measurements of dry matter among organs and lignin, cellulose and sucrose for soybean

The stems, leaves, branches, and pods of 9 soybean plants taken from each treatment, were packed separately into paper bags and dried at 105 °C for 30 min in an electric oven (Sunne, SN-101-3QB, Shanghai, China), and dried at 80 °C until achieving a constant weight, and finally weighed using a balance (Sartorius BSA224S-CW, Beijing, China). After measuring the dry matter weight of each organ, these stems from the 3rd to 5th sections of the soybean were ground with a powder prototype (Hongbang Technology QE-50, Henan, China) to determine contents of lignin, cellulose and sucrose according to previously published methods (Leng et al., 2016; Liu et al., 2016; Wen et al., 2020). All dry leaves from 5 soybean plants taken from each treatment were ground and the sucrose content was measured using the method described by (Leng et al., 2016).

### Grain yield and yield composition

At the mature stage of soybean, 15 soybean plants with uniform growth from each treatment were collected and dried in an air-drying room. These dried plants were used to measure the number of full-pods, non-full-pods and the number of branches per plant, as well as the seed yield per unit area. Similarly, at the mature stage of maize, 15 uniform complete maize cobs from each plot were collected and dried in an air-drying room, which was used to only measure the seed yield per unit area.

### Data analysis

#### Beta and logistic growth models

The dry matter accumulation in each organ (e.g. stems, leaves, branches and pods) and carbohydrates accumulation in leaves and stems for soybean were described assimilate partitioning with beta growth models (Eqn.3-5) (Mao et al., 2018) and logistic growth models (Eqn.6-9) (Zhang et al., 2020). These models could be used to quantitatively characterize the dynamic of biomass accumulation for stems, leaves, branches and pods as well as carbohydrates accumulation in leaves and stems for soybean in the strip intercropping and skip strip monocropping systems.

The beta growth models were described by the following formulas.


(3) $$w=w_{m}\left(1+\frac{t_{e}-t}{t_{e}-t_{m}}\right){\left(\frac{t}{t_{e}}\right)}^{\frac{t_{e}}{t_{e-t_{m}}}}$$

(4) $$\frac{dw}{dt}=c_{m}\left(\frac{t_{e}-t}{t_{e}-t_{m}}\right){\left(\frac{t}{t_{m}}\right)}^{\frac{t_{e}}{t_{e-t_{m}}}}$$

(5) $$c_{m}=w_{m}\frac{2t_{e}-t_{m}}{t_{e}\left(t_{e}-t_{m}\right)}{\left(\frac{t_{m}}{t_{e}}\right)}^{\frac{t_{m}}{t_{e}-t_{m}}}$$

Where *w* (g plant^-1^) is accumulation of dry matter at time *t* (d), *w_m_* (g plant^-1^) is the maximum dry matter accumulation at time *t_e_* (d), *dw/dt* (g plant^-1^ d^-1^) is the daily dry matter growth rate, the maximum growth rate (*c_m_*) of each organ (stems, leaves, branches and pods) is achieved at time *t_m_* (d).

The logistic growth models were expressed by the following formulas.


(6) $$w=\frac{{\text{w}}_{max}}{1+\text{a}\;\text{exp}\left(-\text{bt}\right)}$$

(7) $$v=\frac{\text{dw}}{\text{dt}}=k\text{ab}\frac{\exp\left(-bt\right)}{{\left(1+\text{a}\;\text{exp}\left(-\text{bt}\right)\right)}^{2}}$$

(8) $$c_{max}=\frac{w_{max}*b}{4}$$

(9) $$t_{c}=\frac{1}{\text{b}}\ln\left(\frac{2+\sqrt{3}}{\text{a}}\right)-\frac{1}{\text{b}}\text{ln}\left(\frac{2-\sqrt{3}}{\text{a}}\right)$$

Where *t* is the days after soybean sowing. The *w* (g m^-2^) and *v* (g m^-2^ d^-1^) are the carbohydrates accumulation and the carbohydrates accumulation rate at time *t*, respectively. The *c_max_* is attained when the carbohydrates maximum accumulation (*w_max_*) reaches half. The *t_c_* is the continuous carbohydrates accumulation time. The parameter *b* is the relative accumulation rate and parameter *a* is related to the initial value according to the formula (*a* = (w_max_-w_0_)/w_0_).

### Assimilate partitioning index

The size-dependent changes in soybean dry matter allocation models were divided into stems, leaves, branches and pods by using the method of allochronic analysis (Eqn. 10) (Zhang et al., 2020). The daily allocation was computed as the ratio of the daily growth rate of each soybean organ (e.g. stems, leaves, branches and pods) to the sum of daily growth rates of each soybean organ.


(10) $$AI_{i}=\frac{\frac{dw_{i}}{dt}}{\sum \frac{dw_{i}}{dt}}$$

Where *AI_i_* is the dry matter allocation index for an organ *i* in soybean, $\frac{dw_{i}}{dt}$ represents the daily growth rate for an organ *i* in soybean, *i* stands for stems, leaves, branches and pods, respectively.

### Statistical analysis and graphing

All the data were sequentially collected and sorted out through Excel 2019 software. The statistical software Origin Pro 2020b was applied for two-way analysis of variance (ANOVA), weighted nonlinear fitting and graphing. The significant differences among treatments were separated according to the LSD at *p* ≤ 0.05 and *p* ≤ 0.01. The hypothesis of nonlinear regression model was statistically checked by Chi-square-test and F-test to ensure the correctness of the conclusion based on beta and logical growth curves. The R^2^ in models represented the fitting degree of the curves. The root mean square error (*RMSE*) (Eqn.13) was used to evaluate the effectiveness of the curve models. The determinate coefficient (R^2^) was used to characterize the correlation intensity of LR, LRI and PD, as well as the correlation intensity of LRI with Stem/Leaf, lignin and cellulose.


(13) $$RMSE=\sqrt{\frac{1}{N}\sum {\left(S_{i}-Q_{i}\right)}^{2}}$$

Where *RMSE* is the root mean square error, *N* is the total number of observations, *S_i_* is the fitted values, *Q_i_* is the actual values.

## Results

### Plant height of maize and soybean and maize-crown breadth

**Figure 1:**
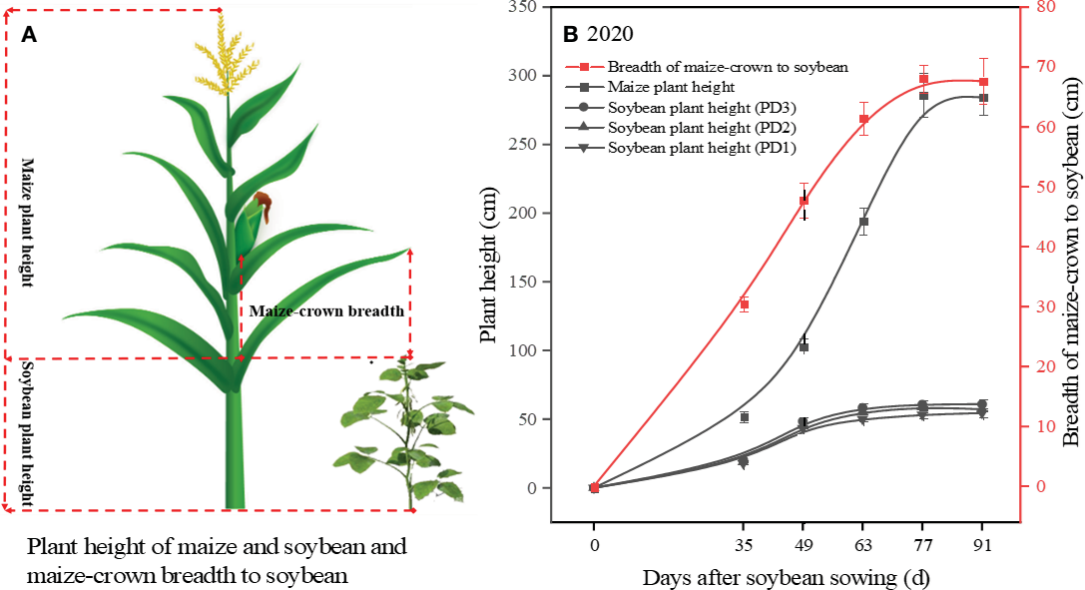
The model of plant height of maize and soybean as well as maize-crown breadth in the strip intercropping system **(A)**. The plant height of maize and soybean response to sowing days [**(B)**, the black solid lines]. The maize-crown breadth response to sowing days [**(B)**, the red solid line]. The black dotted line indicated that maize began to shade soybean **(B)**. Error bars typified represented error of means (± SE).

Plant height of maize and soybean, along with maize-crown breadth, were measured to indirectly assess the impact of maize on the light environment dynamics of soybean in the strip intercropping system (**Figure 1A**). From day 35 after sowing, the height and crown breadth of maize plants exhibited rapid growth, which continued until day 63. During this period, the canopy width of maize surpassed 60 cm, which was the spacing between maize rows and soybean rows, while the height of maize plants reached 194 cm (**Figure 1B**). These findings indicate that the shading effect of maize on soybean gradually intensified from day 35 after sowing and reached a stable level by day 63.

### Grain yield and yield composition

Both planting densities and patterns had significant effects on the grain yield, grain plumpness and branches of soybean, while soybean planting density had no effect on maize yield in the maize-soybean strip intercropping system (**Table 1**). As planting densities increased, the number of full-pods and branches in soybean decreased significantly, whereas the number of non-full-pods increased (**Table 1**). Furthermore, intercropped soybean exhibited significantly lower numbers of full-pods and branches compared to monocropped soybean. Additionally, soybean grain yield in strip intercropping was 55%, 55%, and 59% lower than that in skip strip monocropping at PD1, PD2, and PD3, respectively, over the course of two years (**Table 1**). Thus, the reduced yield of soybean in strip intercropping may be attributed to a lower number of branches and full-pods compared to skip strip monoculture.

**Table 1:** The grain yield and composition of soybean and grain yield of maize response to planting density and pattern in 2019 and 2020.

Years	Treatments		Soybean				Maize
			Grain yield	Full-pods	Non-full-pods	Branches	Grain yield
			g m^-2^	numbers plant^-1^	numbers plant^-1^	numbers plant^-1^	g m^-2^
2019	S-Inter.	PD1	107.06 b	26.67 a	10.33 c	2 a	1105 a
		PD2	119.72 a	23 b	12.67 b	1.33 a	1129 a
		PD3	103.12 b	18.33 c	14.33 a	0.67 a	1148 a
		*P_PD_*	**	**	**	**	n.s.
	S-S-Mono.	PD1	233.76 b	71.67 a	3 c	5.33 a	1080
		PD2	268.21 a	61.33 b	7.67 b	4.33 b	
		PD3	261.46 a	50 c	11.67 a	2.33 c	
		*P_PD_*	*	**	**	**	–
	*P_PP_*		*	**	**	**	–
	P_PD*PP_		**	**	*	*	–
2020	S-Inter.	PD1	102.79 b	26.33 a	11 c	2 a	1222 a
		PD2	114.83 a	25.56 a	13.68 b	1 a	1248 a
		PD3	105.36 b	20.33 b	18 a	0.66 a	1269 a
		*P_PD_*	**	**	**	**	n.s.
	S-S-Mono.	PD1	237.07 b	62.33 a	4.66 c	5 a	1105
		PD2	258.75 a	52.66 b	8 b	3.33 b	
		PD3	251.17 a	44 c	13.25 a	2.67 c	
		*P_PD_*	*	**	**	**	–
	*P_PP_*		**	**	**	**	–
	P_PD*PP_		**	**	*	*	–

Values were means of three biological replicates (SE). Different lowercase letters represented significant difference at *p*< 0.05, the *, ** and n.s. indicated significant levels at *p*< 0.05, *p*< 0.01 and *p* ≥ 0.05, respectively. The S-Inter. and S-S-Mono. were short for strip intercropping and skip strip monocropping, respectively.

### Lodging resistance index (LRI)

**Figure 2:**
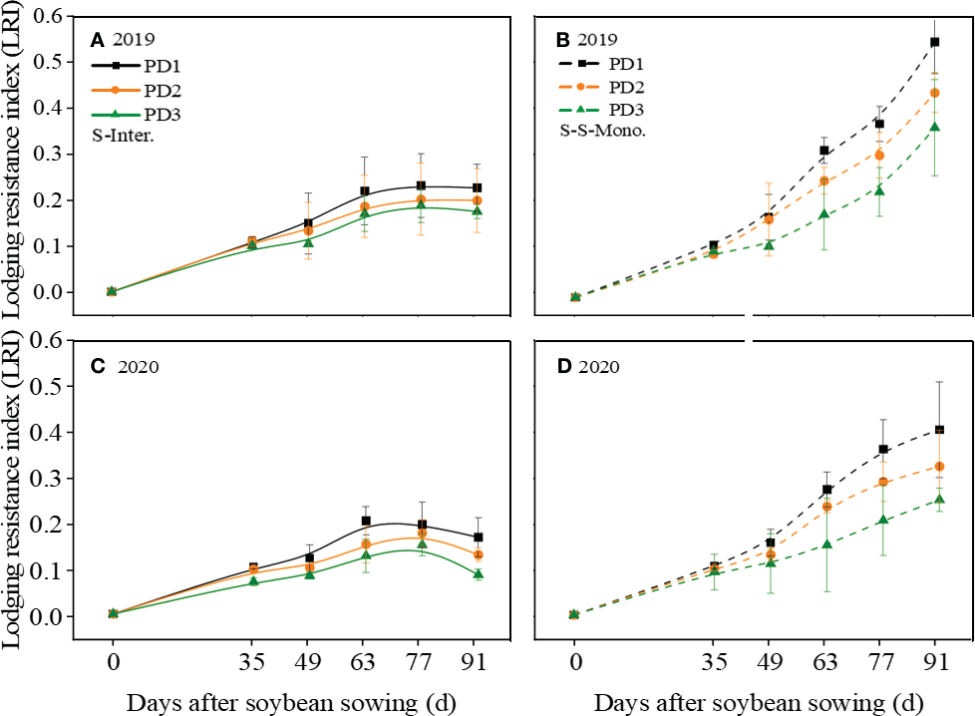
Lodging resistance index (LRI) of soybean response to planting density (PD1, PD2, and PD3) in **(A, C)** strip intercropping (S-Inter.) and **(B, D)** skip strip monocropping (S-S-Mono.) systems in 2019 and 2020. Error bars typiied represented error of means (± SE).

According to **Figure 2**, the LRI (Lodging resistance index) of soybean decreased with the increase of planting density, irrespective of planting patterns. After 49 days of sowing, the LRI of intercropped soybean is lower than that of soybean in monoculture. Furthermore, intercropped soybean reached its maximum LRI at 63 days and then stabilized, followed by a declining trend. In contrast, the LRI of soybean in monoculture continued to exhibit a growth trend until harvest.

### Dry matter growth and daily growth rates for soybean organs

**Figure 3:**
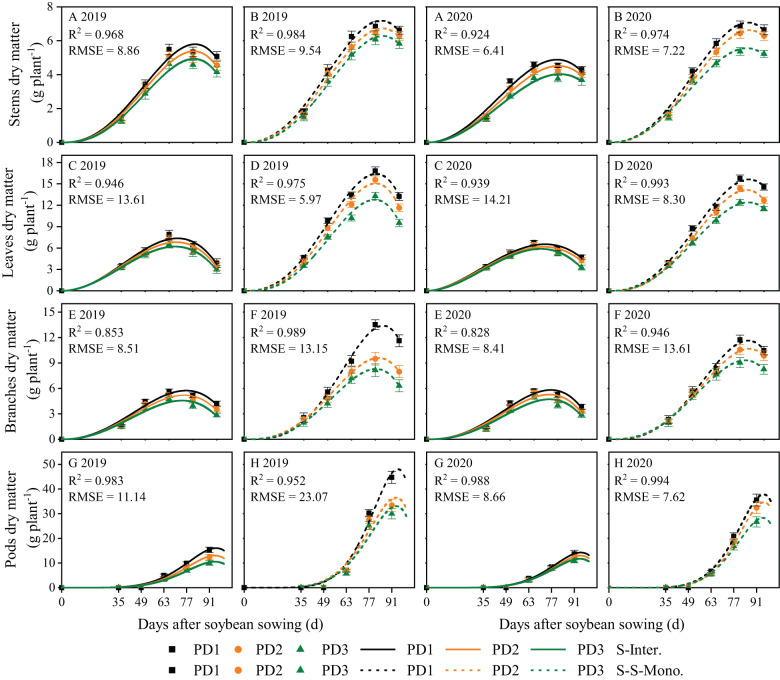
Fitted and observed dry matter growth of soybean stems **(A, B)**, leaves **(C, D)**, branches **(E, F)** and pods **(G, H)** in 2019 and 2020 by beta growth models. The solid line represented the strip intercropping (S-Inter.), and the dotted line represented the skip strip monocropping (S-S-Mono.). The black, orange and green lines represented PD1, PD2 and PD3, respectively. Error bars typified represented error of means (± SE).

**Figure 4:**
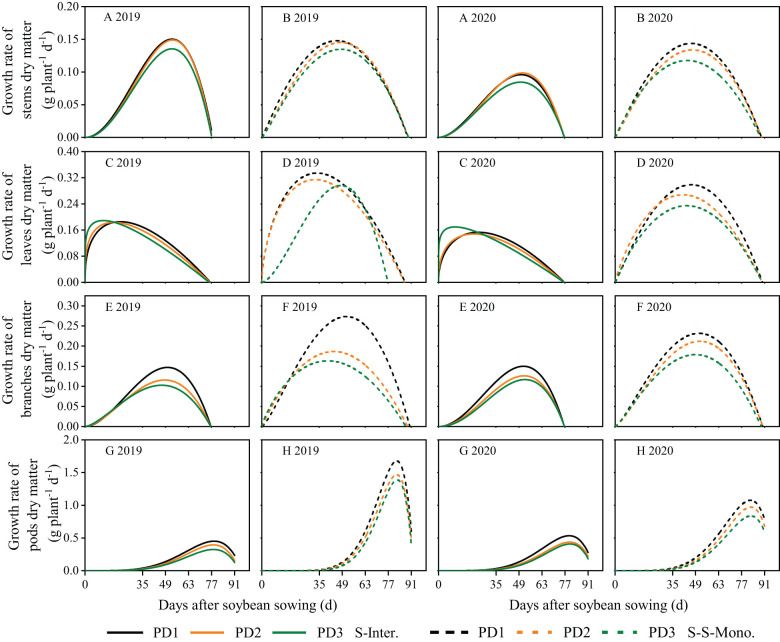
Dry matter growth rates of soybean stems **(A, B)**, leaves **(C, D)**, branches **(E, F)** and pods **(G, H)** in 2019 and 2020. The solid line represents the strip intercropping (S-Inter.), and the dotted line represents the skip strip monocropping (S-S-Mono.). The black, orange and green lines represent PD1, PD2 and PD3, respectively.

The dynamics of soybean organ growth (e.g. stems, leaves, branches, and pods) in strip intercropping and skip strip monocropping were characterized using beta growth curve models due to plant organ shedding and sucrose transfer and transformation in leaves associated with aging (**Figure 3**). The dry matter growth and daily growth rates of each soybean organ exhibited an initial increase followed by a subsequent decrease during the growth and development of soybean (**Figures 3**, **4**). Furthermore, both the dry matter growth and daily growth rates of each soybean organ were significantly lower in strip intercropping compared to skip strip monocropping, and exhibited a significant decrease with increasing planting densities (**Figures 3**, **4**). Additionally, the peak time for both dry matter accumulation and daily growth rates of each soybean organ occurred earlier in strip intercropping compared to the corresponding skip strip monocropping (**Figures 3**, **4**). Furthermore, during the middle and later stages of growth and development, soybeans in strip intercropping exhibited a more pronounced decrease in the growth rate of leaf dry matter compared to other organs, especially at high planting density (**Figure 4**). This phenomenon can be attributed to shading caused by maize, which accelerates soybean leaf senescence.

Planting density, planting patterns, and their interaction significantly influenced the *w_m_*, *c_m_* and *t_e_* of soybean organs (**Supplementary Table 1**). On the one hand, the *w_m_*, *c_m_* and *t_e_* of soybean organs in strip intercropping were lower than those in skip strip monocropping and decreased with increasing planting densities, irrespective of planting patterns (**Supplementary Table 1**). Strip intercropped soybean in higher planting density (PD3) exhibited a decrease of 28% and 11% in *c_m_* and *t_e_*, respectively, compared to the average of low planting density (PD1) over two years (**Supplementary Table 1**). On the other hand, compared to skip strip monocropping, the *t_e_* for soybean organs in strip intercropping was observed 10-14 days earlier, suggesting that interspecific and intraspecific shading reduced the duration of soybean green leaves, leading to decreased soybean dry matter growth and accumulation. Additionally, strip intercropping resulted in a significant advancement in *t_e_* for the growth of stems, leaves, and branches, compared to skip strip monocropping, particularly at high planting density (**Supplementary Table 1**).

### Carbohydrates accumulation and daily accumulation rate for soybean

**Figure 5:**
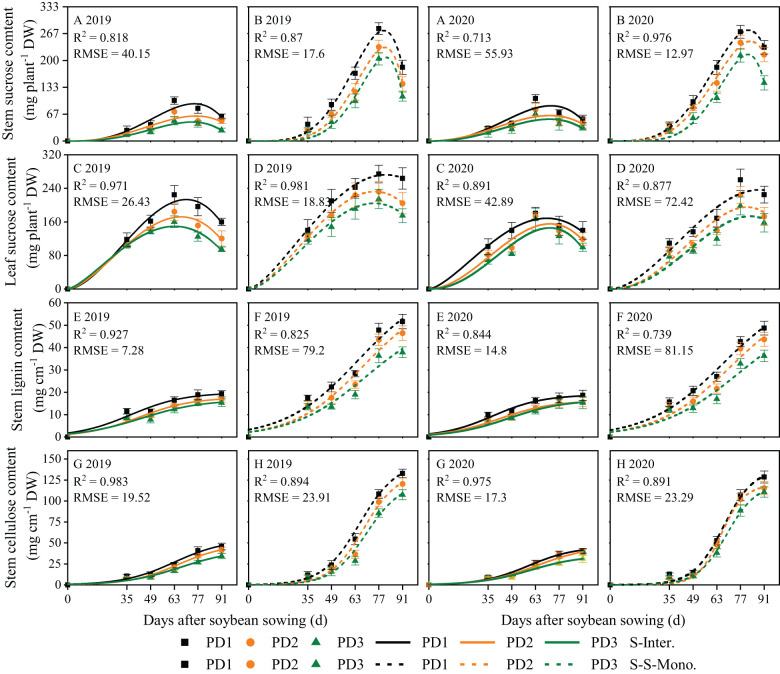
Fitted and observed lignin **(E, F)**, cellulose **(G, H)** and sucrose **(A, B)** accumulation of soybean in 2019 and 2020 by beta **(A–D)** and logistic **(E–H)** growth models. The solid line represents the strip intercropping (S-Inter.), and the dotted line represents the skip strip monocropping (S-S-Mono.). The black, orange and green lines represent PD1, PD2 and PD3, respectively.

**Figure 6:**
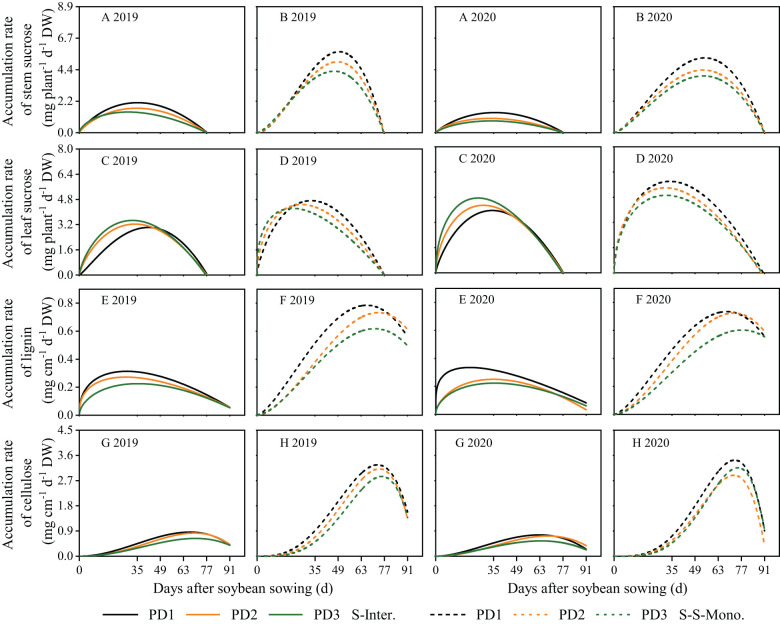
Carbohydrate accumulation rates of stem sucrose **(A, B)**, leaf sucrose **(C, D)**, lignin **(E, F)** and cellulose **(G, H)** in 2019 and 2020. The solid line represents the strip intercropping (S-Inter.), and the dotted line represents the skip strip monocropping (S-S-Mono.). The black, orange and green lines represent PD1, PD2 and PD3, respectively.

Logistic growth curves were effective in accurately fitting the continuous accumulation of lignin and cellulose throughout the entire life cycle of soybean (**Figures 5E–H**). In contrast, the beta growth curves were well-suited for fitting sucrose accumulation in leaves and stems due to its transfer or transformation among organs (**Figures 5A–D**). Sucrose accumulation in leaves and stems exhibited an initial increase followed by a subsequent decrease during the growth and development of soybean, whereas lignin and cellulose accumulation in stems displayed a continuous increasing trend throughout the lifespan of soybean (**Figure 5**). Over the two years of this study, the accumulation of sucrose, lignin, and cellulose and the daily accumulation rate of soybean in strip intercropping were significantly lower than those of corresponding skip strip monoculture and decreased with the increase of planting density (**Figures 5**, **6**). Likewise, the *w_max_*, *c_max_*, *t_e_* and *t_c_* of soybean in the strip intercropping were lower than those in corresponding skip strip monocropping and decreased with increasing planting density (**Supplementary Table 2**). In comparison to the average of low planting density (PD1) over a two-year period, strip intercropped soybean in PD3 exhibited a decrease of 29% in *c_max_* for sucrose (in leaves and stems), lignin, and cellulose (in stems). Furthermore, the *t_c_* of these carbohydrates in intercropped soybean in PD3 decreased by 13%. The peak time for the accumulation of sucrose, lignin, and cellulose in strip intercropped soybean occurred earlier compared to the corresponding skip strip monocropped soybean (**Figure 5**).

Strip intercropped soybean exhibited a greater decrease in sucrose accumulation in leaves and stems compared to sole soybean with skip strip during the middle and later growth stages, particularly at high planting density (**Figures 5A–D**).

Planting density, planting patterns, and their interaction significantly influenced the *w_max_*, *c_max_*, *t_e_* and *t_c_* of lignin, cellulose and sucrose in soybean (**Supplementary Table 2**). Firstly, in the strip intercropping, the *w_max_*, *c_max_* and *t_e_* o values of soybean organs were lower than those in skip strip monoculture, and these values decreased with increasing planting densities (**Supplementary Table 2**). Secondly, the *t_e_* for sucrose and the *t_c_* for lignin and cellulose in the strip intercropping were observably advanced by 16-20 days compared to skip strip monocropping (**Supplementary Table 2**). This observation suggests that shading stress from interspecific and intraspecific interactions reduced the time for sucrose accumulation, consequently affecting the continuous accumulation of lignin and cellulose in soybean (**Figures 5E–H**).

### Assimilate partitioning among soybean organs

**Figure 7:**
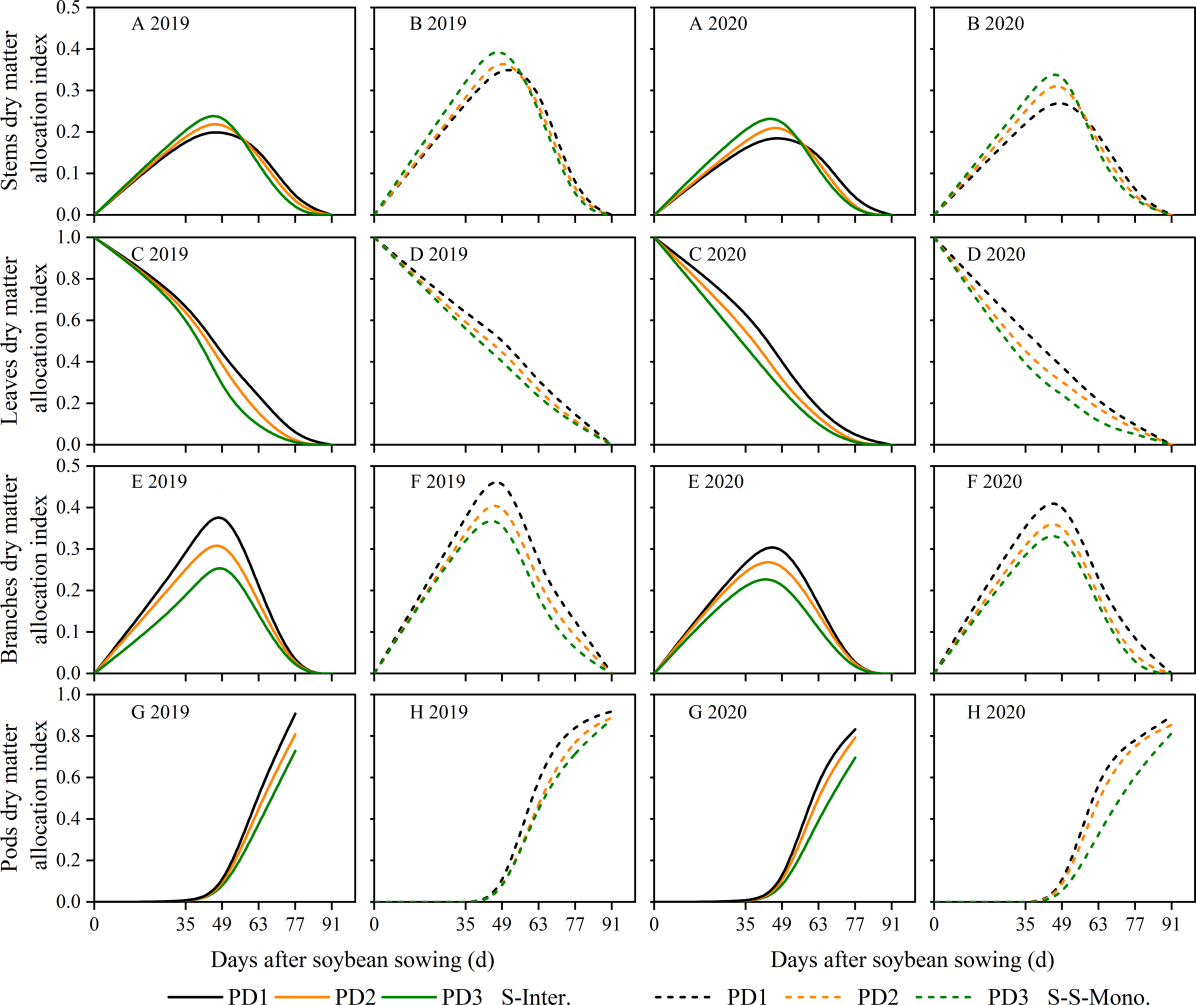
Dry matter allocation index of soybean stems **(A, B)**, leaves **(C, D)**, branches **(E, F)** and pods **(G, H)** in 2019 and 2020. The solid line represents the strip intercropping (S-Inter.), and the dotted line represents the skip strip monocropping (S-S-Mono.). The black, orange and green lines represent PD1, PD2 and PD3, respectively.

The assimilate partitioning index (*AI*) among soybean organs, such as stems, leaves, branches, and pods, can be calculated using the biomass growth rate functions specific to each organ. Our data showed that, during the seedling stage of soybean, a greater proportion of dry matter was allocated to the leaves in terms of time (**Figures 7C, D**). During the middle stage of soybean, a higher proportion of dry matter was allocated to the stems and branches (**Figures 7A, B, E, F**). In the later growth stage, regardless of strip intercropping or skip strip monocropping, a larger portion of dry matter was transferred to the pods to facilitate seed formation (**Figures 7G, H**). When comparing skip strip monocropping with strip intercropping, we observed that the *AI* among soybean organs in strip intercropping was significantly lower than that of sole soybean with skip strip during the same growth period (**Figure 7**), which was closely associated with shading from maize. Furthermore, our research revealed that the *AI* stems showed an increase with increasing planting density during the early stage (0-49 days after soybean sowing), whereas the opposite trend was observed during the later stage (**Figures 7A, B**). Moreover, the *AI* in leaves (**Figures 7C, D**), branches (**Figures 7E, F**) and pods (**Figures 7G, H**) decreased with the increase in planting density in both strip intercropping and skip strip monocropping.

### Stem/leaf AI ratio and stem sucrose/leaf sucrose AR ratio of soybean

**Figure 8:**
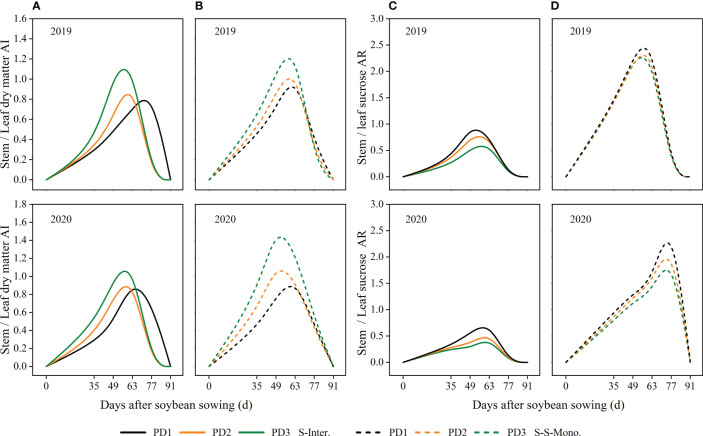
Effects of planting density and pattern on stem/leaf AI ratio (stem/leaf dry matter allocation index ratio) **(A, B)** and stem/leaf sucrose AR ratio (stem/leaf sucrose accumulation rate ratio) **(C, D)** in 2019 and 2020. The solid line represented the strip intercropping (S-Inter.), and the dotted line represented the skip strip monocropping (S-S-Mono.). The black, orange and green lines represented PD1, PD2 and PD3, respectively.

The stem/leaf *AI* ratio increased with increasing planting densities during the early stage (0-49 days after soybean sowing), but exhibited the opposite trend during the later stage of soybean (**Figures 8A, B**). Moreover, the trends of the stem sucrose/leaf sucrose assimilate ratio (*AR*) were consistent with the stem/leaf *AI* ratio (**Figures 8C, D**). Additionally, the value of the stem sucrose/leaf sucrose assimilate ratio (AR) decreased with increasing planting densities in both strip intercropping and skip strip monoculture (**Figures 8C, D**). Furthermore, the value of the stem sucrose/leaf sucrose AR ratio was significantly lower for intercropped soybean compared to soybean in monoculture (**Figures 8C, D**).

### Correlation analysis

**Figure 9:**
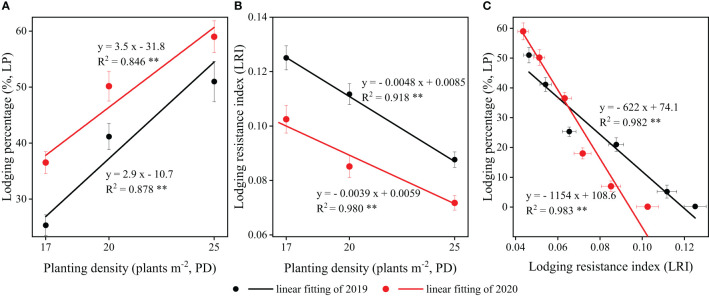
The relationships and correlations between **(A)** planting density (PD) and lodging percentage (LP), **(B)** PD and lodging resistance index (LRI), **(C)** LRI and LP of strip intercropped soybean in 2019 (black) and 2020 (red). The horizontal (X) and vertical (Y) error bars represented standard errors, the * and ** indicated signiicant difference at *p*< 0.05 and *p*< 0.01, respectively.

The two-year field experiments demonstrated that, at the R6 stage, the lodging percentage (*LP*) of strip intercropped soybean exhibited a significant positive correlation with planting densities (**Figure 9A**). Conversely, the lodging resistance index (*LRI*) of strip intercropped soybean displayed a significantly negative correlation with planting densities (**Figure 9B**). Furthermore, the correlations between *LP* and *LRI* for soybean in strip intercropping were significantly negative at both the V3 and R6 stages, regardless of planting densities (**Figure 9C**). Ultimately, the *LRI* serves as a crucial index for measuring the lodging resistance of soybean, irrespective of planting patterns or densities.

**Figure 10:**
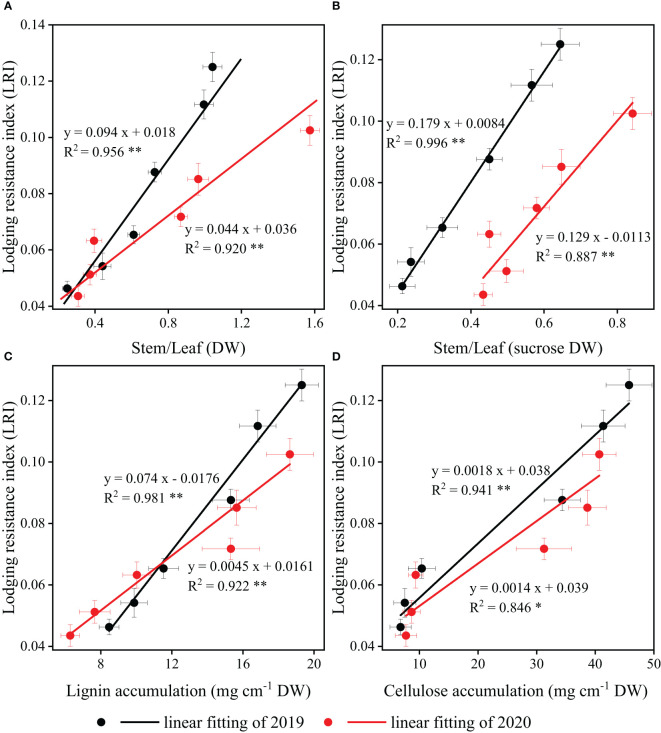
The relationships and correlations between stem/leaf ratio [dry matter **(A)** and sucrose **(B)**] and carbohydrates [lignin **(C)** and cellulose **(D)**] response to lodging resistance index (LRI) of strip intercropped soybean in 2019 (black color) and 2020 (red color). The horizontal (X) and vertical (Y) error bars represented standard errors, the * and ** indicated significant difference at *p<* 0.05 and *p<* 0.01, respectively.

The correlations between *LRI* and stem/leaf ratio (dry matter and sucrose) in strip intercropping were significantly positive, regardless of planting densities (**Figures 10A, B**). Likewise, significant positive correlations were observed between *LRI* and the accumulation of lignin and cellulose in strip intercropped soybean across different planting densities (**Figures 10C, D**).

## Discussion

### LRI response to planting densities and patterns

Lodging is one of the important factors that influences plant morphogenesis and yield formation (Hayes et al., 2017; Lyu et al., 2021). It is affected by various internal and external factors, including plant varieties and planting patterns (Board, 2001; Esechie et al., 2004). The benign external environment was conducive to plant growth and development with stronger stem strength, which in turn lowered lodging risk (Sher et al., 2018). Soybean stem morphology primarily develops during the early growth stages, whereas shading of soybean in the maize-soybean relay strip intercropping system by maize occurs during the seeding stage (Yang et al., 2014; Deng et al., 2016). Reduced photosynthetic active radiation in the soybean canopy within the maize-soybean relay strip intercropping system influenced soybean morphogenesis, resulting in slender and weak stems that are prone to lodging and ultimately leading to a decline in soybean yield (Cooper, 1971; Liu et al., 2017). However, our previous research has found that lodging also occurred in strip intercropped soybeans during the middle and later growth stages when they are shaded (Cheng et al., 2020). A previous study revealed that within the maize-soybean strip intercropping system, the photosynthetically active radiation (PAR) in the soybean canopy experienced a rapid decrease around 40 days after sowing, after which it stabilized around 60 days after sowing, which aligns with our research (**Figure 1B**; Liu et al., 2017). Furthermore, our previous research uncovered that intercropped soybeans at high densities exhibited lodging around 40 days after sowing. As shading intensified, soybeans at medium to low densities gradually encountered lodging in the intercropping system (Cheng et al., 2020). This indicates that soybean lodging is influenced by both interplant shading and intra-plant shading. Soybean in the strip intercropping or higher planting density could increase the shading or self-shading of plants, resulting in higher plant height, lower stem diameter and easier lodging (dos Santos Scholz et al., 2018; Cheng et al., 2020). A significantly negative correlation between *LRI* and *LP* was observed (**Figure 9C**), indicating that *LRI* can serve as an effective index for assessing the lodging resistance of soybean stems. The *LRI* was calculated by the main stem length, the shoot biomass fresh weight and the stem bending force (Hussain et al., 2020a). Previous research has demonstrated that soybean under shaded conditions exhibits elongation of the main stem and reduced stem bending force (Liu et al., 2019; Yang et al., 2020). In this study, we observed a significant negative correlation between *LRI* and plant density (**Figure 9B**), with soybean in monoculture and low plant density exhibiting higher *LRI* compared to soybean in the intercropping system and at high plant density (**Figure 2**).

### The accumulation dynamics of lignin and cellulose in strip intercropped soybean response to planting density

The accumulation of lignin and cellulose in stems is crucial for *LRI* of strip intercropped soybean, and it is influenced by planting densities, planting patterns, and their interaction (Liu et al., 2016; Hussain et al., 2020a). Numerous studies have demonstrated that higher planting density and maize-soybean strip intercropping systems increase the susceptibility of soybean stems to lodging due to the reduced accumulation of lignin and cellulose (Liu et al., 2016; Cheng et al., 2020; Raza et al., 2020). As a precursor of lignin and cellulose synthesis, the accumulation of sucrose in stem affects the lignin and cellulose accumulation in stems (Boerjan et al., 2001; Coleman et al., 2009; Ruan, 2014). A significant positive correlation was found between *LRI* and accumulation of lignin, cellulose and sucrose in stems regardless of planting densities and patterns (**Figure 10**). Beta and logistic growth models were utilized to analyze the accumulation of carbohydrates (e.g., sucrose, lignin, and cellulose), revealing lower contents of these carbohydrates in strip intercropped soybean compared to skip strip monoculture. Furthermore, the contents decreased with increasing planting densities (**Figure 5**). High planting density intensifies the species and interspecific competition for light energy among strip intercropped plants, particularly during the symbiotic period, potentially impacting the distribution of sucrose among soybean organs (Huang et al., 2018; Zhang et al., 2020). The *w_max_*, *c_max_*, *t_e_* and *t_c_* of carbohydrates were affected by planting densities and patterns, and the shade in high planting density inhibited maximum accumulation rate and shortened continuous accumulation time of soybean in both strip intercropping and skip strip monoculture (**Figure 6**; **Supplementary Table 2**). Soybean intercropped at high density exhibited a higher rate of sucrose accumulation in leaves, while the rate of sucrose accumulation in the stems remained consistently low (**Figures 6A, C**). This suggests that shading also influenced the transport of sucrose from the leaves to the stems. In this study, we conducted further analysis of the stem/leaf sucrose accumulation rate ratio (stem/leaf sucrose *AR*) and observed that high planting density promoted sucrose accumulation in leaves while decreasing accumulation in stems, thus impeding the synthesis of lignin and cellulose in the stems (**Figures 8C, D**). Furthermore, several studies have indicated that the relationship between supply and demand of sucrose in different plant organs changes under higher planting density or shade stress. Sucrose synthesis in leaves increases rapidly and is promptly transported to the stems to support plant elongation and growth, thereby capturing more solar energy (Tobias et al., 1999; Cheng et al., 2020). However, at high plant density, the cells in stems exhibited more longitudinal growth and less lateral growth, which aggravated the occurrence of stem lodging (Coleman et al., 2009). Furthermore, it has been demonstrated that optimum nitrogen fertilization boosts soybean stem lodging resistance by modulating the lignin metabolism in the maize-soybean intercropping system (Raza et al., 2023).

### The various organs growth dynamics of strip intercropped soybean response to planting density

The distribution of assimilates among different soybean organs was influenced by planting densities, planting patterns, and their interaction. Numerous studies have shown that shading leads to stem thickness, resulting in stem lodging (Kang et al., 2015; Liu et al., 2016; Song et al., 2016). In this study, the relative content of assimilates in stems to leaves of strip intercropped soybean was closely related to LRI (**Figures 10A, B**). Higher assimilate content in stems is beneficial for maintaining stronger stem strength (Hussain et al., 2020a). Our data indicated that higher planting density not only reduced the growth rate in various organs (**Figure 4**), but also shortened the time to reach maximum dry matter and maximum growth rate for these organs (**Supplementary Table 1**; **Figure 6**) in both strip intercropping and skip strip monoculture. Ultimately, the accumulation of assimilates in various organs reduced (**Figure 3**). Interspecific and intraspecific shading stress significantly restricts the assimilate production of intercropped soybeans, thus, its distribution among various organs determines its stem lodging resistance and ultimately influences yield formation. Light intensity significantly affected the distribution of C-assimilates in the stems and leaves of plants (Hocking and Steer, 1994). We ulteriorly analyzed the ratio of stem assimilate partitioning index to leaf assimilate partitioning index (stem/leaf AI ratio) in this study and observed that increasing planting density accelerated the distribution of assimilates into stems rather than leaves of intercropped soybean during the early growth stage, contributing to enhanced plant height rather than stem diameter of soybean (Cheng et al., 2020; Raza et al., 2020). Increasing the planting density of soybean increased the dry matter allocation index (*AI*) of stems, but it lowered the *AI* of leaves, branches and pods in strip intercropped and skip strip monocropped systems, which affected the final yield formation (**Figure 7**). The most essential reason for the above might be that the unbalanced source-sink relationship of the strip intercropped soybean plants in the vegetative growth period induced by high planting densities and maize shading. In other words, interspecific and intraspecific shading stress resulted in a source-limited soybean in a longer period of symbiosis, which, in turn, leaded to a sink-limited soybean growth. This directly affected the morphogenesis of soybean and yield formation (**Table 1**; Borrás et al., 2004).

## Conclusion

This study presents a novel attempt to investigate soybean lodging resistance in the maize-soybean strip intercropping system by integrating dry matter growth models of soybean organs with carbohydrate accumulation models. The findings demonstrate that shading stress in the intercropping system impedes carbohydrate accumulation and the growth of soybean organs, resulting in decreased sucrose, lignin, and cellulose accumulation in soybean organs. The decrease in the maximum accumulation rate (*c_max_*) and the shortened continuous accumulation time (*t_c_* and *t_e_*) of sucrose, lignin, and cellulose with increasing planting density contributed to this reduction. Similarly, the increase in planting density diminished the maximum dry matter growth rate (*c_m_*) of soybean organs, shortened the time (*t_e_*) for the maximum dry matter growth rate to be reached, and hindered the dry matter growth of soybean organs. Additionally, in the strip intercropping system, high planting density suppressed the transport of sucrose from leaves to stem in soybeans. The combination of these factors resulted in reduced accumulation of lignin and cellulose in the stem and an imbalance in dry matter allocation among soybean organs, leading to stem lodging and yield loss of soybean, especially at high planting densities. The results offer valuable insights into the dynamic changes of dry matter growth in various organs, carbohydrate accumulation in stems, and crop lodging resistance in different planting densities and patterns. This study provides theoretical guidance for the cultivation of crops in intercropping systems with the goal of increasing lodging resistance and yield.

## Data availability statement

The raw data supporting the conclusions of this article will be made available by the authors, without undue reservation.

## Author contributions

LW: Data curation, Investigation, Writing – original draft. BC: Data curation, Investigation, Writing – original draft, Writing – review & editing. TZ: Conceptualization, Resources, Writing – review & editing. SJ: Supervision, Writing – review & editing. RL: Supervision, Writing – review & editing. YG: Investigation, Methodology, Writing – review & editing. CD: Validation, Writing – review & editing. WWY: Validation, Writing – review & editing. ZL: Supervision, Writing – review & editing. AR: Investigation, Writing – review & editing. MX: Investigation, Writing – review & editing. WW: Validation, Writing – review & editing. WL: Funding acquisition, Project administration, Supervision, Writing – review & editing. WYY: Funding acquisition, Project administration, Supervision, Writing – review & editing.
