# Supplementary material for: Quantifying the effects of plant density on soybean lodging resistance and growth dynamics in maize-soybean strip intercropping
Source: Front Plant Sci. 2023 Nov 23;14:1264378. doi: 10.3389/fpls.2023.1264378 (PMC10702229; doi:10.3389/fpls.2023.1264378)
Supplement: Supplementary file 1 [file DataSheet_1.docx]

**Supplementary Tables**

**TABLE S1 |** The maximum dry matter (*w_m_*), maximum growth rate (*c_m_*) and the time (*t_e_*) to reach maximum dry matter between strip intercropping and skip strip monocropping for soybean organs (e.g. stems, leaves, branches and pods) response to planting density in 2019 and 2020.

| Years | Treatments | | Stems | | | | Leaves | | | | Branches | | | | Pods | | |
| --- | --- | --- | --- | --- | --- | --- | --- | --- | --- | --- | --- | --- | --- | --- | --- | --- | --- |
|  |  |  | *W_m_* | *C_m_* | *t_e_* |  | *W_m_* | *C_m_* | *t_e_* |  | *W_m_* | *C_m_* | *t_e_* |  | *W_m_* | *C_m_* | *t_e_* |
|  |  | plants m^-2^ | g plant^-1^ | g plant^-1^d^-1^ | d^-1^ |  | g plant^-1^ | g plant^-1^d^-1^ | d^-1^ |  | g plant^-1^ | g plant^-1^d^-1^ | d^-1^ |  | g plant^-1^ | g plant^-1^d^-1^ | d^-1^ |
| 2019 | S-Inter. | PD1 | 5.8 d | 0.14 a | 75 c | | 7.5 d | 0.17 c | 67 c | | 6.0 c | 0.16 d | 77 c | | 16.0 d | 0.5 c | 86 c |
|  |  | PD2 | 5.2 e | 0.12 ab | 73 cd | | 6.8 de | 0.15 cd | 63 d | | 5.2 c | 0.12 e | 74 cd | | 13.1 e | 0.4 cd | 82 c |
|  |  | PD3 | 4.3 f | 0.11 b | 69 d | | 6.2 e | 0.14 d | 60 d | | 4.8 c | 0.11 e | 73 d | | 10.5 f | 0.3 d | 73 d |
|  |  | SE | 0.3 | 0.01 | 7.2 | | 1.2 | 0.08 | 4.6 | | 1.02 | 0.02 | 5.4 | | 2.6 | 0.01 | 5.6 |
|  | S-S-Mono. | PD1 | 7.8 a | 0.17 a | 89 a | | 16.4 a | 0.35 a | 90 a | | 13.4 a | 0.37 a | 87 a | | 48.1 a | 1.5 a | 98 a |
|  |  | PD2 | 7.2 b | 0.16 a | 85 b | | 15.0 b | 0.33 a | 83 b | | 9.5 b | 0.26 b | 82 b | | 36.5 b | 1.1 ab | 96 ab |
|  |  | PD3 | 6.7 c | 0.13 b | 80 c | | 13.0 c | 0.29 b | 75 c | | 8.3 b | 0.19 c | 81 b | | 33.0 c | 1 b | 94 b |
|  |  | SE | 0.3 | 0.04 | 6.8 | | 2.1 | 0.03 | 5.7 | | 1.3 | 0.04 | 6.8 | | 3.5 | 0.01 | 7.5 |
| 2020 | S-Inter. | PD1 | 5.6 c | 0.13 b | 77 c | | 6.5 c | 0.14 c | 69 c | | 6.1 c | 0.15 c | 78 bc | | 14.3 d | 0.4 c | 85 c |
|  |  | PD2 | 4.8 d | 0.11 c | 74 d | | 6.2 c | 0.13 c | 63 d | | 5.2 cd | 0.12 d | 76 c | | 13.0 de | 0.4 c | 82 c |
|  |  | PD3 | 4.2 e | 0.08 d | 68 e | | 5.8 d | 0.12 c | 59 e | | 4.9 d | 0.08 e | 75 c | | 11.7 e | 0.3 d | 71 d |
|  |  | SE | 0.2 | 0.01 | 3.5 | | 0.81 | 0.005 | 4.6 | | 1.03 | 0.02 | 4.2 | | 1.02 | 0.03 | 6.8 |
|  | S-S-Mono. | PD1 | 7.5 a | 0.15 a | 88 a | | 15.6 a | 0.31 a | 89 a | | 11.7 a | 0.35 a | 85 a | | 37.9 a | 1.2 a | 99 a |
|  |  | PD2 | 6.8 b | 0.14 a | 84 b | | 14.1 b | 0.29 a | 82 b | | 10.7 a | 0.23 b | 84 a | | 34.7 b | 1.1 ab | 96 a |
|  |  | PD3 | 5.8 c | 0.12 bc | 82 b | | 12.4 c | 0.24 b | 76 c | | 9.4 b | 0.2 b | 80 b | | 28.3 c | 0.9 b | 90 b |
|  |  | SE | 0.12 | 0.03 | 2.4 | | 0.86 | 0.012 | 2.63 | | 0.58 | 0.03 | 3.62 | | 2.84 | 0.003 | 2.65 |
| *P* | *P_PD_* | | ** | ns | * | | * | ns | * | | ns | * | ns | | ** | * | ns |
|  | *P_PP_* | | ** | ** | ** | | ** | ** | ** | | ** | ** | * | | ** | ** | ** |
|  | *P_PD*PP_* | | ** | ns | * | | ** | * | ** | | * | ** | ns | | ** | ** | * |

Values were means of three biological replicates (SE). Different lowercase letters represented significant difference at *p* < 0.05, the *, ** and n.s. indicated significant levels at *p* < 0.05, *p* < 0.01 and *p* ≥ 0.05, respectively. The S-Inter. and S-S-Mono. were short for strip intercropping and skip strip monocropping, respectively.

**TABLE S2 |** The maximum carbohydrates (*w_max_*), maximum growth rate (*c_max_*), maximum accumulation time (*t_e_*) and continuous accumulation time (*t_c_*) between strip intercropping and monocropping for sucrose, lignin and cellulose of soybean response to planting densities in 2019 and 2020.

| Years | Treatments | | Sucrose in stems | | | | Sucrose in leaves | | | | Lignin | | | | Cellulose | | |
| --- | --- | --- | --- | --- | --- | --- | --- | --- | --- | --- | --- | --- | --- | --- | --- | --- | --- |
|  |  |  | *W_max_* | *C_max_* | *t_e_* |  | *W_max_* | *C_max_* | *t_e_* |  | *W_max_* | *C_max_* | *t_c_* |  | *W_max_* | *C_max_* | *t_c_* |
|  |  | plants m^-2^ | mg plant^-1^ | mg plant^-1^d^-1^ | d |  | mg plant^-1^ | mg plant^-1^d^-1^ | d |  | mg cm^-1^ | mg cm^-1^d^-1^ | d^-1^ |  | mg cm^-1^ | mg cm^-1^d^-1^ | d |
| 2019 | S-Inter. | PD1 | 126.1 d | 12.1 d | 53 bc | | 212.8 d | 3.7 d | 48 c | | 48.9 d | 0.37 d | 96 d | | 69.9 d | 0.98 c | 98 c |
|  |  | PD2 | 119.4 e | 7.4 e | 50 c | | 177.6 e | 2.9 e | 46 d | | 37.8 e | 0.34 de | 91 de | | 56.7 e | 0.95 c | 92 d |
|  |  | PD3 | 103.9 f | 6.8 e | 42 d | | 148.0 f | 2.6 e | 40 e | | 27.3 f | 0.28 e | 88 e | | 53.7 f | 0.85 d | 87 e |
|  |  | SE | 15.6 | 0.03 | 3.4 | | 6.4 | 0.3 | 2.6 | | 3.7 | 0.013 | 5.6 | | 1.5 | 0.014 | 4.8 |
|  | S-S-Mono. | PD1 | 266.7 a | 44.1 a | 61 a | | 315.2 a | 9.2 a | 57 a | | 116.2 a | 0.78 a | 127 a | | 172.3 a | 3.14 a | 115 a |
|  |  | PD2 | 233.9 b | 40.1 b | 58 ab | | 254.4 b | 7.7 b | 52 b | | 104.3 b | 0.65 b | 115 b | | 166.4 b | 3.07 a | 110 ab |
|  |  | PD3 | 213.9 c | 6.7 c | 55 b | | 219.2 c | 6.6 c | 46 cd | | 92.1 c | 0.58 c | 102 c | | 154.1 c | 2.84 b | 106 b |
|  |  | SE | 20.0 | 0.3 | 6.9 | | 33.6 | 0.4 | 4.8 | | 15.8 | 0.024 | 8.2 | | 5.2 | 0.013 | 8.6 |
| 2020 | S-Inter. | PD1 | 117.2 d | 2.0 c | 50 c | | 205.6 d | 2.9 c | 47 b | | 45.6 c | 0.36 c | 98 d | | 66.9 d | 0.92 d | 94 c |
|  |  | PD2 | 107.8 e | 1.4 d | 48 cd | | 171.2 e | 2.4 d | 44 c | | 36.2 d | 0.27 d | 91 e | | 52.4 e | 0.88 d | 86 d |
|  |  | PD3 | 101.1 f | 1.2 d | 45 d | | 153.6 f | 2.0 e | 41 c | | 25.2 e | 0.22 e | 86 e | | 40.7 f | 0.75 e | 80 e |
|  |  | SE | 13.3 | 0.2 | 4.35 | | 13.3 | 0.4 | 4.3 | | 3.7 | 0.014 | 5.8 | | 2.4 | 0.02 | 4.3 |
|  | S-S-Mono. | PD1 | 275.0 a | 7.7 a | 62 a | | 284.0 a | 9.5 a | 58 a | | 114.9 a | 0.75 a | 125 a | | 165.4 a | 3.04 a | 114 a |
|  |  | PD2 | 241.1 b | 6.8 b | 58 ab | | 260.8 b | 8.6 b | 53 ab | | 102.6 b | 0.70 b | 113 b | | 152.2 b | 2.92 b | 105 b |
|  |  | PD3 | 209.4 c | 6.7 b | 56 b | | 229.6 c | 7.3 c | 47 b | | 98.4 b | 0.62 c | 108 c | | 137.1 c | 2.78 c | 98 c |
|  |  | SE | 7.4 | 0.2 | 3.58 | | 28.32 | 0.3 | 1.65 | | 5.22 | 0.016 | 1.32 | | 2.5 | 0.014 | 3.6 |
| *P* | *P_PD_* | | ** | * | ns | | ns | ** | * | | ns | ** | * | | * | ** | * |
|  | *P_PP_* | | ** | ** | ** | | * | ** | ** | | ** | ** | ** | | ** | ** | ** |
|  | *P_PD*PP_* | | ** | ** | * | | * | ** | ** | | ** | ** | ** | | ** | ** | ** |

Values were means of three biological replicates (SE). Different lowercase letters represented significant difference at *p* < 0.05, the *, ** and n.s. indicated significant levels at *p* < 0.05, *p* < 0.01 and *p* ≥ 0.05, respectively. The S-Inter. and S-S-Mono. were short for strip intercropping and skip strip monocropping, respectively.

**Supplementary Figures**

**FIGURE S1 |** Daily maximum and minimum air temperatures of soybean from sowing to harvest in 2019 (A) and 2020 (B) in Chongzhou, Sichuan, China. The dark orange represented the daily maximum air temperature, the light orange represented minimum air temperature.


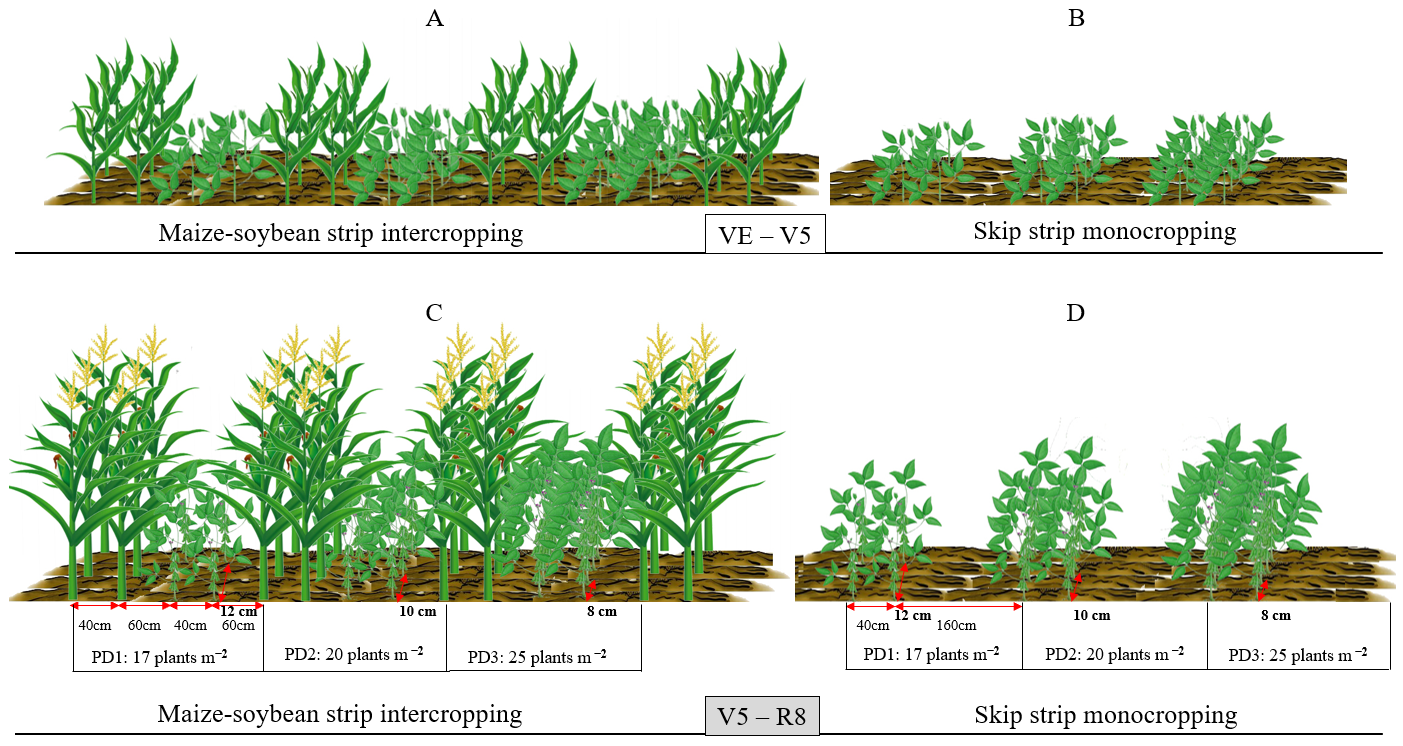


**FIGURE S2 |** The patterns of maize soybean strip intercropping (A and C) and skip strip monocropping (B and D) in 2019 and 2020. Three planting densities (PD1, PD2, and PD3) with a number of 17 plants m^−2^, 20 plants m^−2,^ and 25 plants m^−2^. Soybean was not shaded by maize during VE - V5 (A), while the shading of maize on soybean occurred during V5 - R8 (C).
